# Classifying neovascular age-related macular degeneration with a deep convolutional neural network based on optical coherence tomography images

**DOI:** 10.1038/s41598-022-05903-7

**Published:** 2022-02-09

**Authors:** Jinyoung Han, Seong Choi, Ji In Park, Joon Seo Hwang, Jeong Mo Han, Hak Jun Lee, Junseo Ko, Jeewoo Yoon, Daniel Duck-Jin Hwang

**Affiliations:** 1 Department of Applied Artificial Intelligence, Sungkyunkwan University, Seoul, Korea; 2 RAON DATA, Seoul, Korea; 3 Department of Medicine, Kangwon National University Hospital, Kangwon National University School of Medicine, Chuncheon, Gangwon-do South Korea; 4 Seoul Plus Eye Clinic, Seoul, Korea; 5 Kong Eye Center, Seoul, Korea; 6 Department of Ophthalmology, Hangil Eye Hospital, 35 Bupyeong-daero, Bupyeong-gu, Incheon, 21388 Korea; 7 Lux Mind, Incheon, Korea; 8 Department of Ophthalmology, Catholic Kwandong University College of Medicine, Incheon, Korea

**Keywords:** Retinal diseases, Medical imaging, Machine learning

## Abstract

Neovascular age-related macular degeneration (nAMD) is among the main causes of visual impairment worldwide. We built a deep learning model to distinguish the subtypes of nAMD using spectral domain optical coherence tomography (SD-OCT) images. Data from SD-OCT images of nAMD (polypoidal choroidal vasculopathy, retinal angiomatous proliferation, and typical nAMD) and normal healthy patients were analyzed using a convolutional neural network (CNN). The model was trained and validated based on 4749 SD-OCT images from 347 patients and 50 healthy controls. To adopt an accurate and robust image classification architecture, we evaluated three well-known CNN structures (VGG-16, VGG-19, and ResNet) and two customized classification layers (fully connected layer with dropout vs. global average pooling). Following the test set performance, the model with the highest classification accuracy was used. Transfer learning and data augmentation were applied to improve the robustness and accuracy of the model. Our proposed model showed an accuracy of 87.4% on the test data (920 images), scoring higher than ten ophthalmologists, for the same data. Additionally, the part that our model judged to be important in classification was confirmed through Grad-CAM images, and consequently, it has a similar judgment criteria to that of ophthalmologists. Thus, we believe that our model can be used as an auxiliary tool in clinical practice.

## Introduction

Neovascular age-related macular degeneration (nAMD), a degenerative macular disease, is among the leading causes of blindness in elderly people over 50 years of age in developed countries^1,2^. nAMD can be classified into one of the three subtypes^3^: (i) typical nAMD, (ii) polypoidal choroidal vasculopathy (PCV), and (iii) retinal angiomatous proliferation (RAP). The different subtypes of nAMD could have different post-injection treatment responses and disease prognosis^4^. PCV has been considered a variant form of nAMD, is more prevalent among Asians; however, its distinct characteristics have led to the recognition that PCV might be a different disease entity from typical nAMD^5–7^. Disease course and response to PCV treatment have been shown to differ from typical AMD forms: because of variants of PCV or massive macular hemorrhage, long-term outcomes of PCV treatment are still controversial, but better responses to photodynamic therapy have been reported^8,9^. Retinal angiomatous proliferation (RAP) is another subtype of nAMD, characterized by retinal-retinal or retinal-choroidal anastomoses, and has a poor functional prognosis because it is likely to respond poorly to treatment compared to other subtypes of nAMD^10–13^.

Multimodal imaging, including fluorescein angiography (FA) or indocyanine green angiography (ICGA), has been demonstrated to be effective in accurately diagnosing nAMD^3,7,14^. Accurate diagnosis is essential for establishing an appropriate treatment strategy and accurately predicting a patient's prognosis. Among these modalities, optical coherence tomography (OCT) is noninvasive and is often used to evaluate the structural abnormalities associated with nAMD, and is used as an adjunct to these angiography methods^7,14^.

Recent advances in deep learning technologies such as Convolutional Neural Networks (CNNs) have made it possible to classify nAMD or other macular disorders using only OCT images^15–21^. In the prior work^19^, it has been reported that distinguishing RAP from PCV using a deep learning model and OCT images could be possible, and hence such a deep learning approach could support ophthalmologists in distinguishing RAP from PCV.

The present study proposes and evaluates a comprehensive diagnosis system for nAMD, rather than a simple binary classification between PCV and RAP. We propose a deep learning model that can classify all the subtypes of nAMD, including typical AMD with type 1 or type 2 choroidal neovascularization (CNV) using OCT images alone. The performance of the proposed model was compared with that of an ophthalmologist. Additionally, the structural differences of these nAMD subtypes were investigated by using gradient-weighted class activation mapping (Grad-CAM) heatmaps to visualize the specific features determined by the proposed model.

## Results

In this study, we conducted a study based on 4749 OCT images from 397 participants. The mean ages of the normal and nAMD groups were 64.66 ± 8.41 and 75.40 ± 8.74 years, respectively. Detailed information on the data used in this study is presented in Table 1.

**Table 1:** Baseline characteristics of patients who had undergone macular OCT.

	Normal	Typical AMD	neovascular AMD		
			RAP	PCV	Total
Image, no	2125	806	863	955	2624
Patients, no	50	120	106	121	347
Age, yrs. (SD)	64.66 (8.41)	80.35 (6.21)	80.35 (6.21)	71.07 (8.33)	75.40 (8.74)
**Gender, no (%)**					
Male	23 (27.06)	20 (18.69)	20 (18.69)	82 (67.21)	102 (44.54)
Female	62 (72.94)	87 (81.31)	87 (81.31)	40 (32.79)	127 (55.46)
**Eye, no. (%)**					
Right	44 (51.76)	55 (51.40)	55 (51.40)	64 (52.46)	119 (51.97)
Left	41 (48.24)	52 (48.60)	52 (48.60)	58 (47.54)	110 (48.03)

*OCT* optical coherence tomography, *neovascular AMD* neovascular age-related macular degeneration, *RAP* retinal angiomatous proliferation, *PCV* polypoidal choroidal vasculopathy, *SD* standard deviation.

### Model performance

We conducted experiments to compare three different CNN models (VGG-16, VGG-19, and Resnet) with two custom layers. Table 2 shows the details of each model, such as the number of parameters used and the best accuracy on the test set (920 images) among the fivefold cross validations. As shown in Table 2, the VGG-16 based model with four fully connected layers and three dropout layers showed the highest accuracy (87.4%) on the test set.

**Table 2:** Comparative results of our deep learning models (VGG-16, VGG-19 and Resnet based Model).

Base model	Custom layer	# of parameters	Accuracy (%)
VGG-16	4 Fully connected layer + 3 Dropout layer (LeakyRelu)	27,636,388	87.4%
	Global average pooling	14,716,740	86.1%
VGG-19	4 Fully connected layer + 3 Dropout layer (LeakyRelu)	32,946,084	87.3%
	Global average pooling	20,026,436	86.8%
Resnet	4 Fully connected layer + 3 Dropout layer (LeakyRelu)	75,021,668	85.4%
	Global average pooling	23,572,996	83.0%

### Performance comparison with ophthalmologists

**Figure 1:**
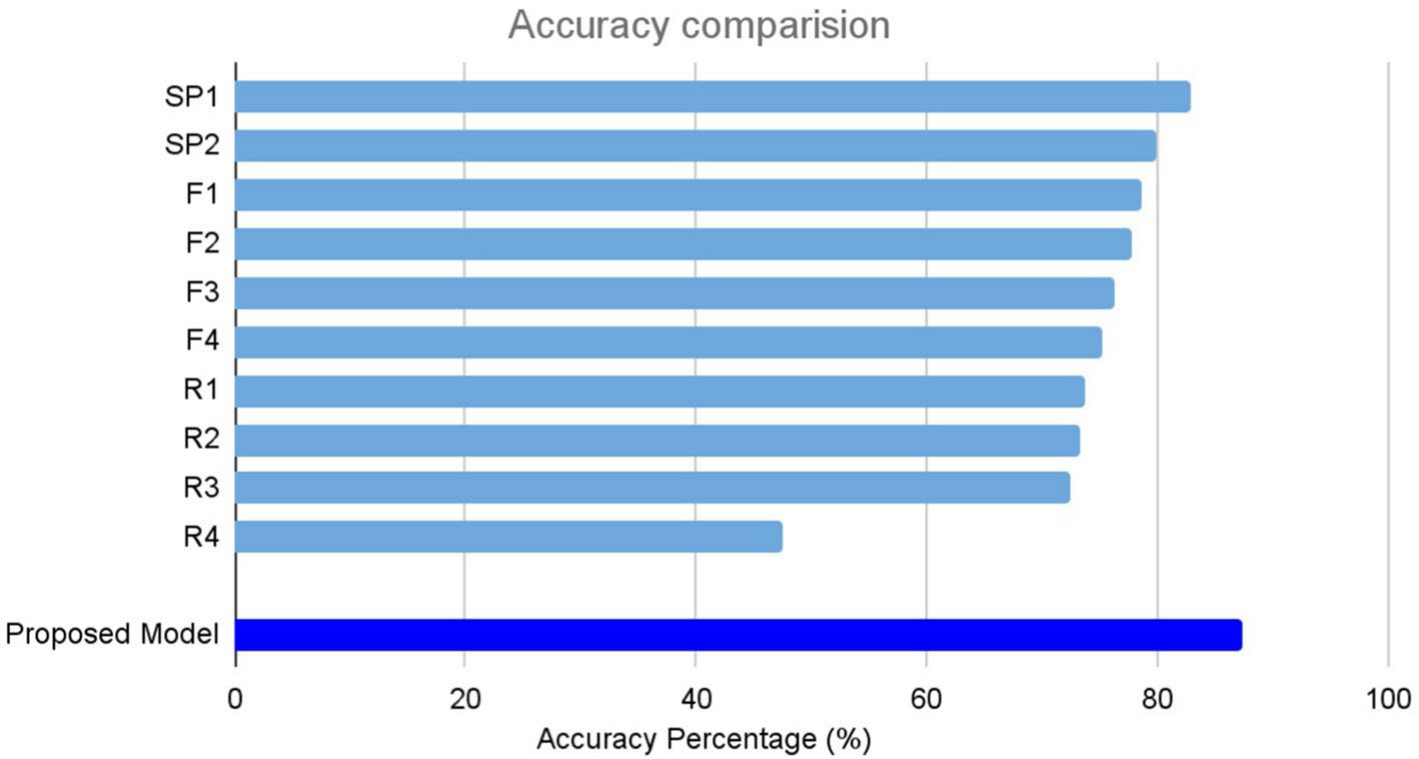
Comparison of classification performance with model and 10 ophthalmologists. The classification accuracy of the model was 87.4%, the highest performance compared to ten ophthalmologists. Ophthalmologists consisted of two retina specialists (RE), four retina fellows (F) and four residents (R).

The performance comparison between the proposed model (i.e., VGG-16 with four fully connected and three dropout layers) and ten ophthalmologists is shown in Fig. 1. The classification accuracies of the ten ophthalmologists ranged from 47.4 to 82.8%. Of the ten ophthalmologists, two retina experts with more than 10 years of clinical experience at an academic ophthalmology center showed the highest classification accuracy of 82.8% and 79.9%, respectively, which was lower than our model’s accuracy (87.4%).

**Figure 2:**
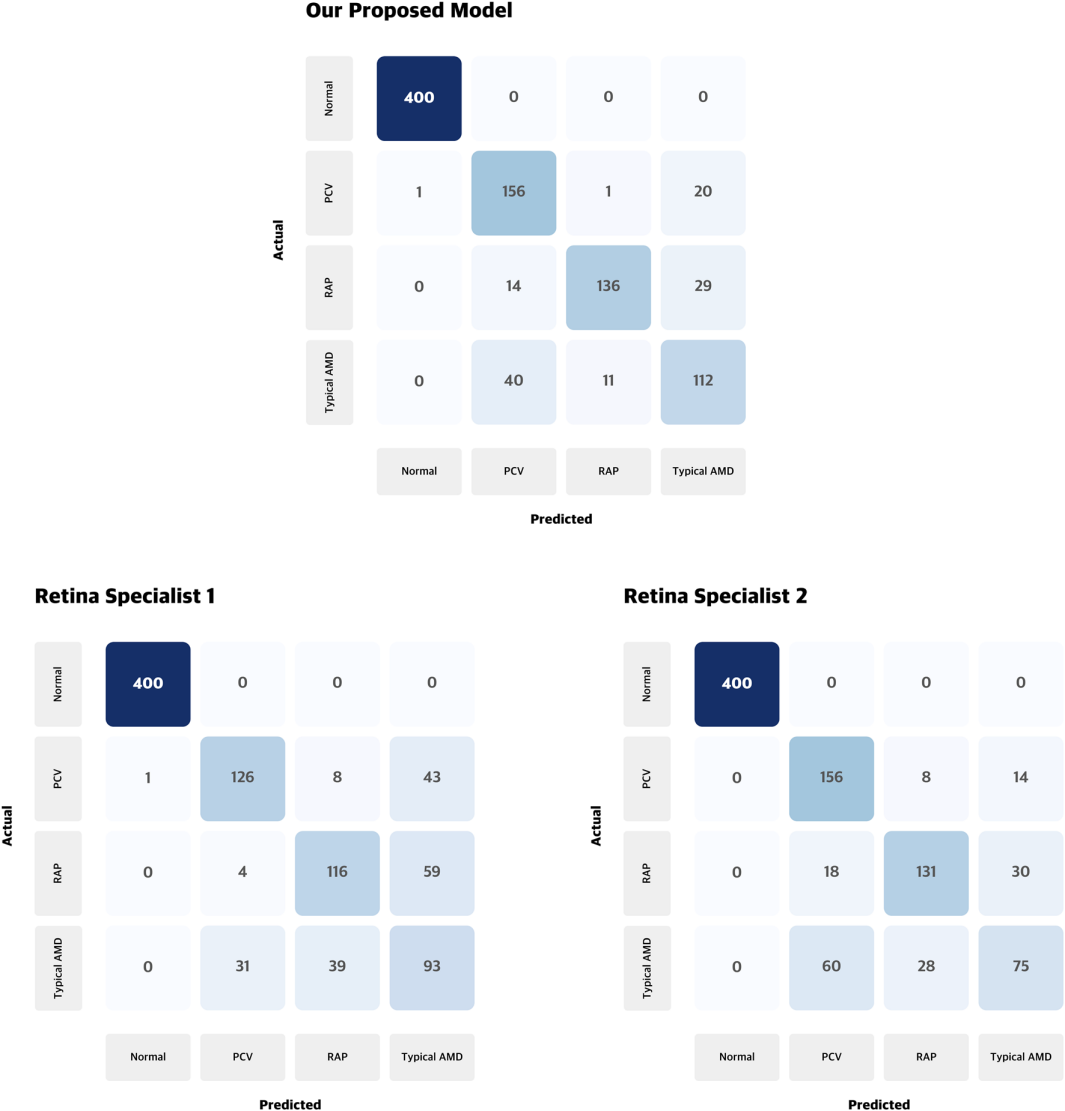
Confusion matrixes of the two retina specialists and the proposed model. Two retinal specialists showed the highest classification accuracy among 10 ophthalmologists, and the classification accuracy was 79.9% and 82.8%, respectively. Our proposed model recorded 87.4% classification accuracy for the same test dataset, higher performance than the two retinal specialists.

We also measured the kappa coefficients between the two retina experts and our proposed model. The kappa coefficient between the two experts was 0.70. The kappa coefficients between the model and the two experts were 0.75 and 0.77, respectively. This suggests that the decision-making criteria for the nAMD subtype classification of our proposed model are similar to those of experts. In addition, looking at various performance measures, as shown in Table 3, we found that the model performed better than the two experts in precision, recall, and F1-score. Furthermore, as shown in the confusion matrix for the test set in Fig. 2, the overall classification performance of the proposed model is higher than that of the two retina specialists.

**Table 3:** Classification results of comparative performance of Our model and 2 retina specialist.

Class	Class	Precision	Recall	F1-Score
Our model	Normal	1.00	1.00	1.00
	PCV	0.74	0.88	0.80
	RAP	0.92	0.76	0.83
	Typical AMD	0.70	0.69	0.69
Specialist 1	Normal	1.00	1.00	1.00
	PCV	0.78	0.71	0.74
	RAP	0.71	0.65	0.68
	Typical AMD	0.48	0.57	0.52
Specialist 2	Normal	1.00	1.00	1.00
	PCV	0.67	0.88	0.76
	RAP	0.78	0.73	0.76
	Typical AMD	0.63	0.46	0.53

*OCT* optical coherence tomography, *neovascular AMD* neovascular age-related macular degeneration, *RAP* retinal angiomatous proliferation, *PCV* polypoidal choroidal vasculopathy.

As a result of the experiment, we identified 15 cases that were correctly classified by the two retina experts but were incorrectly categorized by six or more of the remaining eight ophthalmologists, suggesting that professional experience with retinal disorders is required. Of these 15 cases, the proposed model correctly classified 14 cases, indicating that the model could play a subsidiary role in the diagnosis of nAMD among normal, typical AMD, PCV, and RAP.

### Visualizing the classification process of our model using Grad-CAM

**Figure 3:**
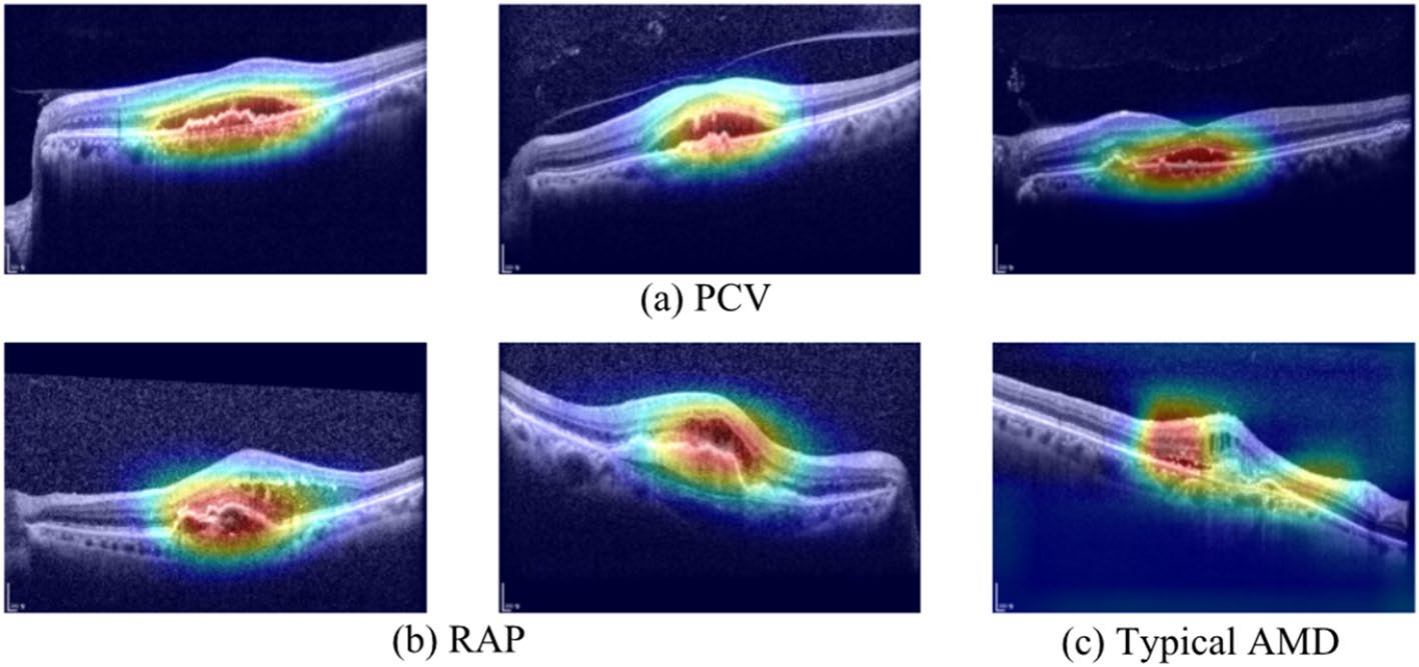
Visualization of 6 cases of AMD subtypes (3 PCV cases, 2 RAP cases, 1 Typical AMD cases) using Grad-CAM on our deep learning model. (**a**) Three PCV cases, (**b**) Two RAP cases, (**c**) One Typical AMD case. The high intensity area (red and yellow color) reflects the area of interest to our model. The six images used in this figure were correctly classified by two retina specialists and our model, but six or more ophthalmologists were misclassified. The corresponding Grad-CAM image shows that our model actually showed the similar criteria for classifying AMD subtypes.

In this study, we used gradient-weighted class activation mapping (Grad-CAM), a technique that visualizes the region where a deep learning model recognizes the important classification features. The representative heat maps generated by Grad-CAM are shown in Fig. 3. The images used in Fig. 3 are Grad-CAM images of three PCV cases, two RAP cases, and one typical AMD case. These images were correctly classified by two retina experts and our model, but more than six out of eight ophthalmologists classified them incorrectly. The areas highlighted in the heat map are those that are recognized as important for classification. These areas are similar to those that ophthalmologists usually examine when diagnosing patients with nAMD. This implies that our model can classify nAMD subtypes with clinically meaningful criteria.

## Discussion

In this study, we presented a deep learning model and investigated its performance in distinguishing several subtypes of nAMD using SD-OCT images. Our model not only classified between the normal and nAMD groups, but also further classified the nAMD group into PCV, RAP, and typical AMD, which showed a performance comparable to that of ophthalmologists. In addition, our model correctly classified the images with incorrect readings by more than half of the ophthalmologists participating in the test, demonstrating that our model could provide additional help in nAMD classification by ophthalmologists.

Several studies^15–18^ have tried to (1) differentiate macular disease from normal macula, and (2) classify retinal diseases into various macular diseases such as AMD, diabetic retinopathy, and epiretinal membrane using OCT. Going one step further, there have been attempts to classify AMD using fundus photographs and OCT. Using the two modalities, Chou et al. differentiated PCV from nAMD with EfficientNet and multiple correspondence analysis^22^. Moreover, Xu et al. classified nAMD, Dry AMD, PCV, and normal groups using deep CNN networks^23^. However, no report has been on a deep learning model that can classify nAMD in more detail subtypes, such as typical AMD, PCV, and RAP, using a single modality. In addition, our study compared the classification accuracy of the proposed model with 10 ophthalmologists who have various clinical experiences. Through a detailed division of nAMD into specific subtypes, we believe our work would be helpful in predicting the treatment responses and prognoses.

This model could also assist the ophthalmologist in interpreting the OCT images. For 15 images that most ophthalmologists misclassified (more than 6 out of 8 remaining ophthalmologists), the proposed model correctly classified 14 (93.3%), two retina experts classified them correctly. In addition, high kappa coefficients were found not only between the two retina experts, but also between our model and each retina expert. This suggests that the proposed deep learning model can help non-retina experts classify subtypes of nAMD, a task that requires the involvement of experienced retinal experts.

In the results of Grad-CAM, the highlighted area was mainly the foveal region of the retina. This implies that our model mainly looked at foveal lesions, not the parafoveal or perifoveal regions when distinguishing subtypes of nAMD. Note that the foveal region is the area at which ophthalmologists mainly look at when classifying nAMD using OCT. When the model plays an auxiliary role in the diagnosis by ophthalmologists in actual clinical practice, if not only the reading results of the model but also visualization tools such as Grad-CAM are presented, more reliable results and interpretations can be provided by ophthalmologists. Interestingly, unlike the retina, the choroidal region was not significantly considered in the proposed nAMD classification model. The choroid was barely highlighted in the heat map using Grad-CAM. Several studies have shown that thinning of the choroid is characteristic of RAP^14,24,25^, whereas PCV involves thickening of the choroid^26–28^. The Grad-CAM results of our study suggest that choroidal thickness was not an essential feature used by the proposed model to discriminate subtypes of nAMD. Future studies should investigate whether choroidal findings influences the development of deep learning models capable of discriminating the subtypes of nAMD.

To optimize the performance of classifying subtypes of nAMD from a limited number of OCT images, we applied several deep learning methodologies. First, we applied transfer learning, a method of reusing knowledge of a source domain to solve a target task (classifying subtypes of nAMD in our work). In this study, a pre-trained model based on the ImageNet dataset was trained on the OCT data. As a result, the model with transfer learning showed a higher performance score than the model without transfer learning. Second, data augmentation was used to reduce overfitting by increasing the variance of the OCT dataset. Data augmentation is a technique commonly performed in deep-learning-based image classification tasks. It is usually performed based on simple parametric transformations, such as rotation, zoom in–out, and resizing images. To ensure that the newly generated SD-OCT images maintained the disease-related information of the original OCT images, we applied the following data augmentation process. First, the images were moved vertically and horizontally. Next, we flipped the training set images horizontally.Third, we rotated the training set images at an angle between 0–15 degrees. By applying these various learning methodologies, we were able to prevent overfitting on the limited OCT dataset and generate a deep learning model with high classification performance.

Our study has several limitations. First, we investigated the performance of the model using only one OCT image. In clinical practice, ophthalmologists typically examine multiple OCT images of the same patient to make a comprehensive diagnosis. For the diagnosis of nAMD, combining multiple images may be better than basing the judgment on only one isolated OCT image. Second, the variety and number of OCT images available were limited. In addition, all images were acquired using a single OCT device. In future studies, external validation with OCT devices sourced from different manufacturers will be necessary. However, the dataset was sufficient to demonstrate the feasibility of our proposed deep learning model to distinguish the subtypes of nAMD using OCT images. Despite these limitations, our model successfully classified the nAMD subtypes based on a single SD-OCT image, suggesting the possibility of future study directions toward developing nAMD diagnostic models using multiple SD-OCT images.

In summary, we developed a deep learning model that performed well in distinguishing between several subtypes of nAMD using only OCT images without a segmentation algorithm. Automation of the classification process using this model may support ophthalmologists in differentiating nAMD subtypes. We believe that this study forms the basis for further studies to develop accurate OCT-based deep learning models with high performance for detecting nAMD and for classifying several macular diseases.

## Methods

### Ethics statement

This study was conducted in accordance with the 1964 Helsinki Declaration. The Ethics Committee of Hangil Eye Hospital approved the research protocols and their implementation. The committee waived the requirement for obtaining informed consent, given that this was a retrospective observational study of medical records and was retrospectively registered.

### Data collection and labeling

We analyzed the records of patients who visited the Hangil Eye Hospital between January 2014 and January 2020. We used SD-OCT (Heidelberg Spectralis; Heidelberg Engineering, Heidelberg, Germany) images of normal healthy participants and patients with nAMD. Among the 347 patients enrolled at the outpatient clinic during that period, 120 had typical AMD, 106 had RAP, and 121 had PCV. Additionally, 50 participants were assigned to the normal healthy group. All typical AMD, RAP, and PCV cases were diagnosed by independent retina specialists using fundus photographs, FA, ICGA, and OCT images. One eye per patient was selected for this study, with one visit per patient. The FA-/ICGA-based classification of nAMD was performed by two retina specialists (DDH and JSH) who reviewed all images obtained by OCT, FA, and ICGA multimodal imaging. In cases of disagreement, a third retina specialist (JMH) assessed the discrepancy and discussed the case with other specialists. All discrepancies were resolved by consensus.

**Figure 4:**
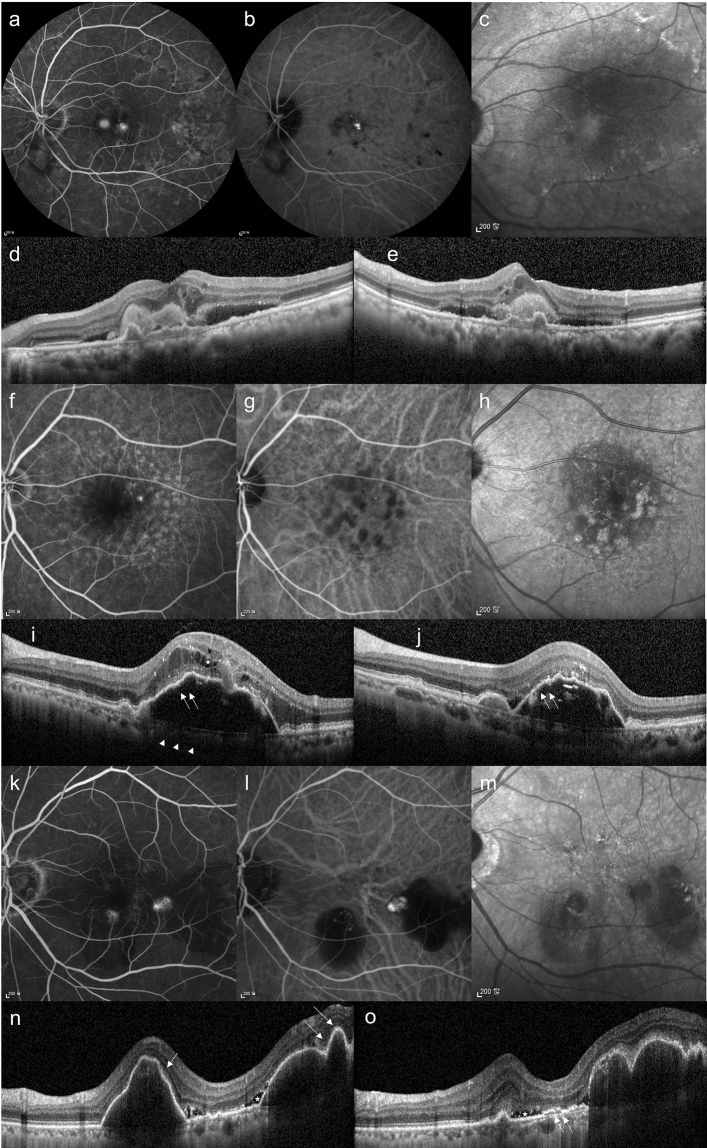
Representative cases of typical AMD (**a**–**e**), RAP (**f**–**j**) and PCV (**k**–**o**). Typical AMD. (**a**) FA, (**b**) ICGA, (**c**) IR, and (**d**,**e**) OCT images. The OCT images (**d**,**e**) show typical features of AMD with type 2 CNV. RAP. (**f**) FA, (**g**) ICGA, (**h**) IR, and (**i**,**j**) OCT images of an eye with RAP. The OCT images (**i**,**j**) show typical features of RAP: a thin choroid (arrowheads), intraretinal cyst like fluid accumulation (asterisks), and trapezoid-shaped RPED (double arrows). PCV. (**k**) FA, (**l**) ICGA, (**m**) IR, and (**n**,**o**) OCT images of an eye with PCV. The OCT images (**n**,**o**) show irregular RPE elevation with double-layer sign (double arrow), subretinal fluid (asterisks), and sharp-peaked or steeper dome-shaped RPEDs or notches (arrows). *FA* fluorescein angiography, *ICGA* indocyanine green angiography, *IR* infrared reflectance, *OCT* optical coherence tomography, *CNV* choroidal neovascularization, *PCV* polypoidal choroidal vasculopathy, *RAP* retinal angiomatous proliferation, *RPE* retinal pigment epithelium, *RPED* retinal pigment epithelial detachment.

PCV was diagnosed based on the presence of polypoidal lesions with or without branching vascular networks^19^. Cases that exhibited retinal-retinal or retinal-choroidal anastomoses were classified as type 3 neovascularization (RAP)^19^. The remaining patients who were not diagnosed with either PCV or RAP were classified as having typical nAMD with type 1 or type 2 choroidal neovascularization (CNV). Figure 4 shows representative cases of each subtype of nAMD. Our analysis excluded cases that showed other potentially conflicting retinal pathologies, such as central serous chorioretinopathy, diabetic retinopathy, and branch retinal vein occlusion.

### SD-OCT dataset collection

In this study, we used only central volume scans consisting of 25 scan images.

**Figure 5:**
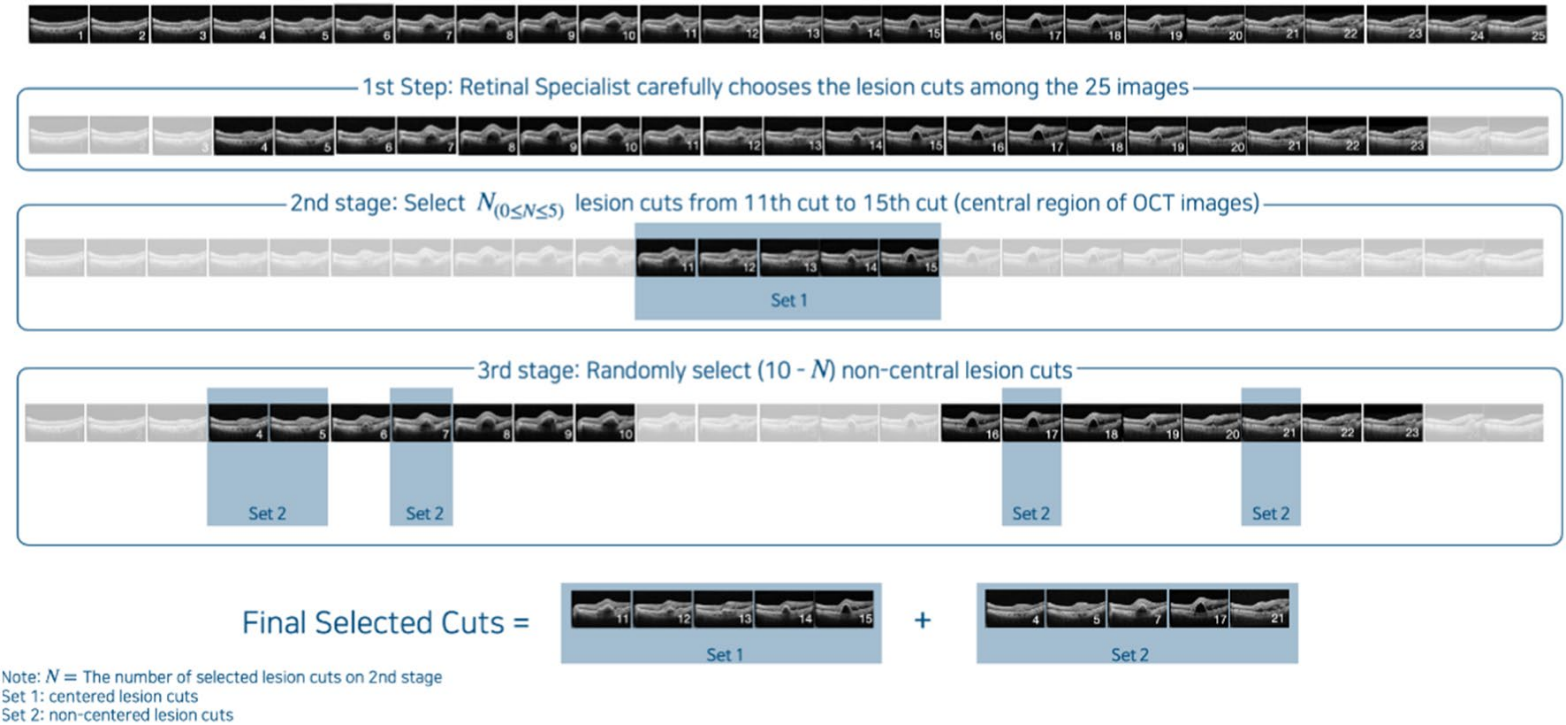
Entire process of extracting lesion cuts from 25 SD-OCT scan images. Initially, Retina expert (DDH) extracted lesion cuts from the 25 SD-OCT images for patient. Next, we selected N (i.e., 0 ≤ N ≤ 5) lesion cuts between the 11th and 15th central regions. Finally, we randomly selected 10-N non-centered lesion cuts. Therefore, up to 10 images were selected per patient for this study.

Figure 5 illustrates the overall process of extracting lesion cuts from the 25 scan images. As shown in Fig. 5, DDH first selected all lesion cuts from the 25 SD-OCT images for the patient. He considered an SD-OCT image as a lesion cut if any of the following findings were observed: (1) Subretinal fluid (SRF), (2) Intraretinal fluid (IRF) or intraretinal cyst, (3) Drusen or Irregular RPE elevation with double-layer sign, (4) Pigment epithelial detachment (PED), and (5) Subretinal hyperreflective material. The lesion cuts were carefully selected by checking all 25 SD-OCT images of each patient. After that, we selected the N (i.e., 0 ≤ N ≤ 5) lesion cuts that were included in the central region (between the 11th and 15th cuts). We then randomly selected 10-N non-centered lesion cuts that are located in non-centered lesion cuts (including the parafoveal or perifoveal area) that are located between the 1st and 10th cuts or between the 16th and 25th cuts. If the number of non-central lesion cuts is less than 10-N, all non-central lesion cuts are selected. Therefore, up to 10 images were selected per patient in this study.

### Data preprocessing

First, we cropped whole SD-OCT scan images to RGB images of size 490 × 764 for using only lesion cuts in training the deep learning model. Then, we down-sampled the images into 224 × 224 RGB images. This is because using the entire SD-OCT scan images (490 × 764) takes up too much memory, and the 224 × 224 RGB format is widely used in deep learning models for image classification. The entire dataset was randomly split into a training set (80%) and a test set (20%). The training set consisted of 3,829 images (normal, 1725; PCV, 777; RAP, 684; typical AMD, 643) of 316 patients (Normal: 40, PCV: 96, RAP: 84, typical AMD: 96). The test set consisted of 920 images (normal: 400, PCV: 178, RAP: 179, typical AMD: 163) of 81 patients (normal, 10; PCV, 25; RAP, 22; typical AMD, 24). Here, the same patient's data do not belong to either the training or test sets at the same time.

Building a robust classification model requires a large amount of training data. However, owing to the lack of training data, we applied data augmentation during the training phase. Data augmentation has been demonstrated as a promising way to increase the performance of classification tasks^29^. For each image, we generated images that were shifted, zoomed in/out, rotated, and flipped. Augmentation was not applied to the test data (i.e., 920 images).

### Model architecture

**Figure 6:**
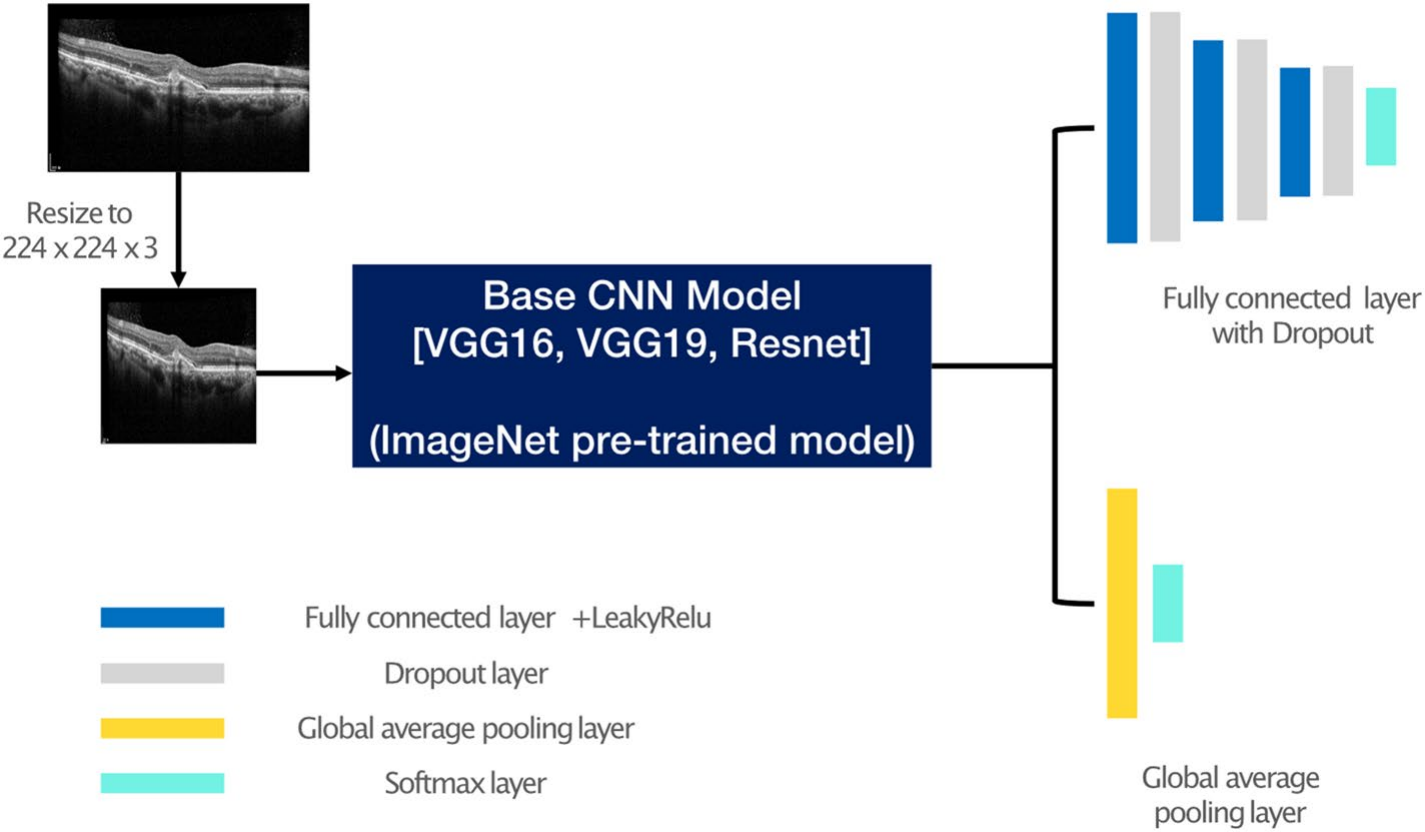
Network architecture of the modified CNN-based models (VGG-16, VGG-19, Resnet) in our study. Using 3 CNN-based pre-trained models (VGG-16, VGG-19, Resnet) and 2 last classification layers (fully connected layer with dropout and global average pooling), a total of 6 models were generated and used in this experiment.

To adopt an accurate image classification architecture in deep learning, we evaluated well-known CNN models, namely, VGG16^30^, VGG19^30^, and Resnet^31^. To improve the classification performance, we also applied transfer learning with the ImageNet dataset^32,33^. In transfer learning, a pre-trained model using a large dataset, such as ImageNet^34^ was used. Note that ImageNet has 15 million annotated images with 1000 classes^34,35^. By transferring the ImageNet-based pre-trained model to our SD-OCT images, we could obtain an accurate and robust model. In our experiment, we found that using transfer learning with the pre-trained model could increase the accuracy by 2.1%. To customize the model, we replaced the fully connected layers of the original CNN models (VGG-16, VGG-19, and Resnet) with two custom settings: (1) four fully connected layers and three dropout layers with Leaky ReLU as an activation function, and (2) a global average pooling layer. An illustration of the proposed model architecture is shown in Fig. 6.

To fairly compare all the possible model architectures, for example, VGG 16 with a global average pooling, VGG19 with 4 fully-connected layers/3 dropout layers/leaky Relu, all the models were trained with the same hyperparameters. The batch size of the model was 64, the loss function was the categorical cross entropy with Adam optimization, and the learning rate was 0.0001.

### Experiment setup

To train and evaluate the proposed model, we conducted a fivefold cross-validation. We split the entire SD-OCT dataset into training (80%) and a test (20%) set. Next, we divided the training data into five folds. Among the five folds, one fold plays the role of a validation set, while the other folds are used in training. We repeated this five times until we validated all folds. Thus, the proposed model was validated for each fold. Finally, the performance of the final model was evaluated using a test set (920 images).

To compare the performance of our proposed model with that of ophthalmologists, ten ophthalmologists (two retina specialists, four retina fellows, four residents) were asked to classify 920 SD-OCT scanned images that were the same as the test set, which was used to evaluate our model's performance.

### Gradient-weighted class activation mapping

We used Grad-CAM to visualize potential pathological areas in OCT images^36^. To visualize the critical regions of the image for the classification of the target label, Grad-CAM extracts the gradient of the target labels with respect to the feature map of the convolutional layer to generate a heat map showing the critical area during the classification process.

### Statistical analysis

To measure the performance of the model, precision, recall, F1-score, and accuracy were calculated. Cohen's kappa coefficient was used to assess the agreement level between the two retina specialists and the proposed model. Cohen’s Kappa coefficients were calculated using Scikit-learn, which is a well-known Python library.

## Data Availability

The data are not available for public access because of patient privacy concerns, but are available from the corresponding author upon reasonable request.
